# Hsp90 N- and C-terminal double inhibition synergistically suppresses Bcr-Abl-positive human leukemia cells

**DOI:** 10.18632/oncotarget.14324

**Published:** 2016-12-28

**Authors:** Chun Chen, Yingting Zhuang, Xianling Chen, Xiaole Chen, Ding Li, Yingjuan Fan, Jianhua Xu, Yuanzhong Chen, Lixian Wu

**Affiliations:** ^1^ Deptartment of Pharmacology, School of Pharmacy, Fujian Medical University (FMU), Fuzhou, China; ^2^ Institute of Materia Medica, School of Pharmacy, Fujian Medical University (FMU), Fuzhou, China; ^3^ Fuijan Key Laboratory of Natural Medicine Pharmacology, School of Pharmacy, Fujian Medical University (FMU), Fuzhou, China; ^4^ Fujian Institute of Hematology, Union Hospital, FMU, Fuzhou, China; ^5^ Deptartment of Biopharmaceutics, School of Pharmacy, Fujian Medical University (FMU), Fuzhou, China

**Keywords:** Hsp90, amino terminal domain, carboxyl terminal domain, Bcr-Abl, imatinib–resistant, leukemia stem cell

## Abstract

Heat shock protein 90 (Hsp90) contains amino (N)–terminal domain, carboxyl(C)-terminal domain, and middle domains, which activate Hsp90 chaperone function cooperatively in tumor cells. One terminal occupancy might influence another terminal binding with inhibitor. The Bcr-Abl kinase is one of the Hsp90 clients implicated in the pathogenesis of chronic myeloid leukemia (CML). Present studies demonstrate that double inhibition of the N- and C-terminal termini can disrupt Hsp90 chaperone function synergistically, but not antagonistically, in Bcr-Abl-positive human leukemia cells. Furthermore, both the N-terminal inhibitor 17-AAG and the C-terminal inhibitor cisplatin (CP) have the capacity to suppress progenitor cells; however, only CP is able to inhibit leukemia stem cells (LSCs) significantly, which implies that the combinational treatment is able to suppress human leukemia in different mature states.

## INTRODUCTION

Hsp90 is a molecular chaperone that activates over 200 client proteins involved in multiple survival signaling pathways, which are targets for chemotherapeutic strategies [1–4]. Hsp90’s role as a center regulator has made it a promising candidate for drug development [1, 5].

Hsp90 has three highly conserved domains, including N- and C-terminal domains and a middle domain. Both N- and C- terminal domains of Hsp90 can initiate chaperone activity by binding to cochaperones or ATP [6]. Geldanamycin (GA) and 17-AAG are specific N-terminal inhibitors under phase II clinical trials. The middle domain interacts with client proteins and co-chaperones. To date, there is no middle domain specific inhibitor. CP is a selective C-terminal-specific agent [7, 8].

The three domains of Hsp90 interact with each other. By applying direct biochemical approaches, Soti C*, et al*. unveiled that the C-terminal ATP-binding chaperone site is opened by occupancy of the N-terminal site [6, 7]. These results implicated that a 17-AAG occupied N-terminal domain would promote chaperone function of the C-terminal domain. Moreover, Hsp90 C-terminal inhibitor novobiocin (NB) disrupted the binding of Hsp90 to the N-terminal inhibitor l, whereas the N-terminal inhibitor could not affect NB-Sepharose binding. Additionally, the binding of NB to the C-terminal domain inhibits N-terminal chaperone function by blocking nucleotide binding. In contrast to dual function of NB, CP was able to inhibit the C-terminal function specifically.

The Bcr-Abl kinase encoded by *bcr-abl* fusion gene is implicated in the pathogenesis and chemotherapeutic resistance of CML. Bcr-Ab activates many signal transduction pathways, including Crkl, NF-kB, and STAT pathways [9–13]. Since Bcr-Abl protein is one of the known clients of Hsp90 [14–19], disruption of the chaperone functions of Hsp90 may potentially block signal transduction pathways activated by Bcr-Abl. Imatinib is a highly effective therapy for CML by inhibiting Bcr-Abl tyrosine kinase activity. However, relapses have been observed and are much more prevalent in patients with advanced disease. ABL kinase mutation and the insensitivity of CML LSCs to imatinib are major reasons for CML relapse [20–23]. Thus, the development of novel approaches distinct to ABL kinase inhibition is urgent.

**Figure 7:**
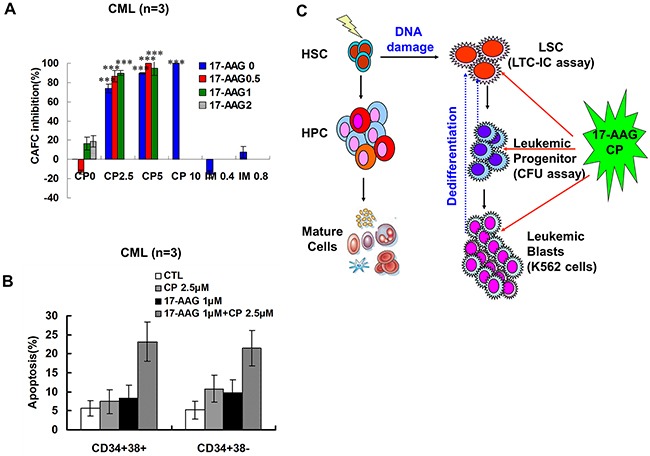
Effects of 17-AAG and CP on CML primitive and committed progenitors **A**. 17-AAG and CP suppressed the self-renewal of primitive progenitors (LTC-ICs). After treating with 17-AAG and CP at the indicated concentrations for 24 h, MNCs from CML bone marrow were examined by LTC-ICs assay. The percent inhibition of LTC-ICs proliferation in 17-AAG and CP treated group relative to untreated controls was shown (CML, n = 3). **B**. Representative data for CML primitive and CML committed progenitor apoptosis. After treating with 17-AAG and CP at the indicated concentrations for 24 h, CML CD34^+^CD38^-^ primitive and CD34^+^CD38^+^ committed progenitors were incubated with Annexin V-FITC solution. The Annexin V positive cells were evaluated by FACS. **C**. The scheme of the origins of the LSCs and the effects of 17-AAG+CP on LSCs via eradicated leukemia cells at different mature states.

LSCs may originate from mutant hematopoietic stem cells, dedifferentiated leukemia committed progenitors, and mature leukemia cells that reacquire self-renewal capability [24–27] (Figure 7C). Thus, the strategy of eradicating these three origins of LSCs together may cure leukemia.

Until now, there have been approximately 13 Hsp90 inhibitors undergoing clinical trials (https://clinicaltrials.gov/). Given that biochemical studies demonstrated the interaction between N- and C-terminal Hsp90 domains, this study aims to explore the final comprehensive biological functions of combination therapy of the N-terminal inhibitor and the C-terminal inhibitor in Bcr-Abl positive leukemia cells, which will provide evidence for clinical chemotherapy approaches in the future. Because NB disrupts both C- and N-terminal function, we used selective C-terminal inhibitor CP in this study. These studies demonstrate that cotreatment with N- and C-terminal Hsp90 inhibitors in a synchronous manner can disrupt Hsp90 chaperone function synergistically in Bcr-Abl-positive human leukemia cells, which successfully retard the Bcr-Abl initiating signal pathway. Furthermore, either 17-AAG or CP has the capacity to suppress leukemia progenitor cells; however, only CP is able to inhibit leukemia stem cells significantly, which implies the combination treatment is better than single therapy treatments and the former may suppress human leukemia cells in different mature states at the same time.

## RESULTS

### Hsp90 N-terminal inhibitor 17-AAG and C-terminal inhibitor CP interact with Hsp90 and inhibit its ATPase activity

**Figure 1:**
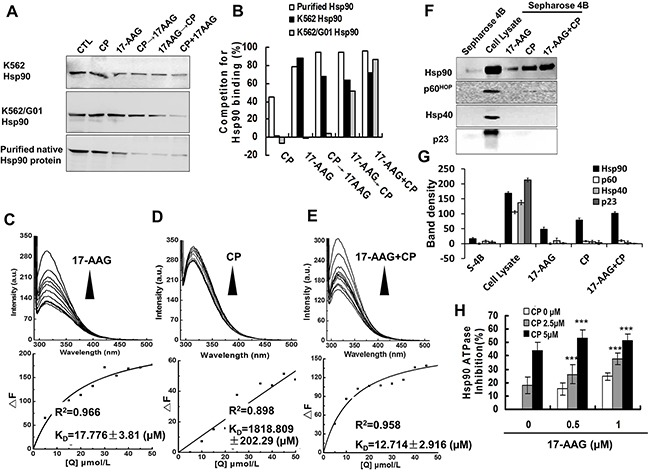
17-AAG and CP had affinity to Hsp90 and suppressed Hsp90 ATPase activity in vitro **A**. 17-AAG and CP could compete for Hsp90 binding from bio-GA by single treatment or co-treatment: 17-AAG (1 μM), CP (5 μM), 17-AAG+CP for 30 min, CP 30 min→17-AAG 30 min, 17-AAG 30 min→CP 30 min. Hsp90 was fromK562 or K562/G01 leukemic cells expressing Bcr-Abl, or purified Hsp90 protein. **B**. Quantification of competition for Hsp90 binding tested by western blot. **C-E**. The fluorescence quenching spectra of NHsp90 with 17-AAG, CP, or 17-AAG+CP. Upper panel: NHsp90 concentration was maintained at 5 μM, and the ratio of drugs vs NHsp90 ranged from 1:1 to 10:1. Lower panel: The variation tendency of Δ*F*. **F**. 17-AAG and CP influence Hsp90 and its co-chaperones connected to Sepharose 4B. Cell lysates were mixed with 17-AAG-Sepharose 4B, CP-Sepharose 4B, or 17-AAG+CP-Sepharose 4B. The levels of bound Hsp90 and its co-chaperones in the pull down proteins were analyzed by western blotting. **G**. Band density of Sepharose 4B pull down assay. **H**. The ATPase activity of NHsp90 was suppressed by Hsp90 inhibitors.

To investigate whether Hsp90 N-terminal and C-terminal inhibitors will interact with each other in binding Hsp90, we first did competitive binding assays using a biotinylated GA (biotin-GA) probe (Figure 1A-1B). Incubation of immunoprecipitated Hsp90 from K562 chronic leukemia cells or imatinib resistant chronic leukemia cells K562/G01 with 17-AAG or CP interfered with the binding of Hsp90 to biotin-GA modestly, whereas the sequential or simultaneous co-treatment with 17-AAG and CP inhibited the interaction more significantly than single agent treatment. Thus, *in vitro* co-treatment also has more effect than a single agent treatment.

Next, we tested the binding of 17-AAG and CP to Hsp90 by the fluorescence quenching of Hsp90. When His-tagged N-terminal Hsp90 (NHsp90) was incubated with increasing concentrations of 17-AAG or/and CP, the intrinsic fluorescence of NHsp90 gradually decreased (Figure 1C-1E). The K_D_ values were 17.776±3.81 μM for 17-AAG, 1818.809±202.29 μM for CP, and 12.714±2.916 μM for 17-AAG+CP, respectively. Additionally, when another C-terminal inhibitor NB was used instead of CP (Supplementary Figure 1), we obtained similar results. These results suggested that 17-AAG and CP could interact with Hsp90 synchronously.

Third, we tested the physical binding of Hsp90 and 17-AAG or/and CP using 17-AAG or/and CP-conjugated Sepharose 4B beads. 17-AAG or/and CP-conjugated Sepharose pull-down assay confirmed that 17-AAG and CP could interact physically with Hsp90 directly. However, the cochaperones, p60^HOP^, p23 and Hsp40, could not be pulled down (Figure 1F and 1G), which implied that the Hsp90 inhibitor disrupted the chaperone function of Hsp90.

Fourth, we examined the ATPase activity of Hsp90 influenced by 17-AAG and CP. 17-AAG and CP suppressed Hsp90 ATPase activity in a concentration-dependent manner. The inhibition rate of Hsp90 ATPase activity was up to 50% after 17-AAG 0.5 μM + CP 5 μM treatment (Figure 1H). Again, these results suggested that co-treatment has more effect on the Hsp90 chaperon function than single agent treatment.

### Combinational treatment with 17-AAG and CP disrupts Hsp90 chaperone function efficiently in leukemia cells

**Figure 2:**
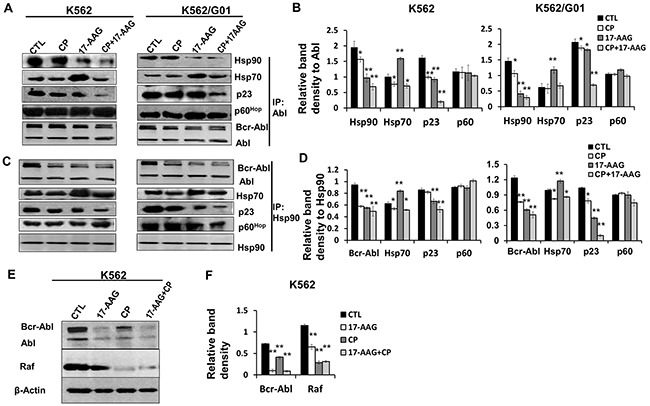
17-AAG and CP suppressed the complex formation of Bcr-Abl with Hsp90 and co-chaperones **A**. Treatment with 17-AAG, CP, or 17-AAG+CP for 6 h changed the protein components in chaperone complexes formed with Bcr-Abl. Lysates of K562 and K562/G01 cells were immunoprecipitated by anti-Abl mAb. Co-precipitation of molecules of the Hsp90 chaperone complexes was analyzed by immunoblotting. Abl served as input control. **B**. Band densities were measured and normalized to Abl in the assay of co-immunoprecipitated with Abl. *p<0.05, ** p<0.01 vs control. **C**. Treatment with 17-AAG, CP, or 17-AAG+CP for 6 h changed the protein components in chaperone complexes Bcr-Abl/Hsp90, and Hsp90/Hsp70. Cell lysates of K562 and K562/G01 were immunoprecipitated by anti-Hsp90 mAb. Co-precipitation of molecules of the Hsp90 chaperone complexes was analyzed by immunoblotting. Hsp90 served as input control. **D**. Band densities were normalized to Hsp90 in the assay of co-immunoprecipitated with Hsp90. *p<0.05, ** p<0.01 vs control. **E**. Hsp90 inhibitors suppressed Hsp90 chaperone function in K562 cells. Client molecules (Bcr-Abl, Raf) of Hsp90 were determined in K562 cells which treated by17-AAG, CP, or 17-AAG+CP for 24 h. **F**. The relative band densities of Bcr-Abl and Raf were normalized to β-actin. ** p<0.01 vs control. Note: 17-AAG: 17-AAG 1 μM; CP: CP 5 μM.

To assess how Hsp90 N-terminal and C-terminal inhibitor interfere with multi-chaperone complexes, we determined the levels of essential components associated with Hsp90 and Bcr-Abl,such as Hsp70, p23 and p60^Hop^, in K562 and K562/G01 cells (Figure 2A).

We carried out coimmunoprecipitation experiments to investigate the effects of 17-AAG and/or CP on the Hsp90 chaperone function (Figure 2A and 2B). Leukemia cells were treated with 17-AAG 1 μM, CP 5 μM, or 17-AAG 1 μM + CP 5 μM for 6 h. Figure 2A demonstrated that treatment with 17-AAG, CP, or 17-AAG+CP significantly decreased the level of Hsp90 that were coprecipitated with Bcr-Abl.

Alternatively, we further examined whether antibodies to Hsp90 could immunoprecipitate Bcr-Abl and whether 17-AAG, CP, or 17-AAG+CP inhibited the complex. Cell lysates from K562 and K562/G01 cells treated with 17-AAG 1 μM, CP 5 μM, or 17-AAG 1 μM + CP 5 μM for 6 h were precipitated using an anti-Hsp90 antibody (Figure 2C and 2D). Figure 2 shows that there was a moderate decrease in the association of Bcr-Abl with Hsp70 as well as Hsp90 in the cells treated with CP. Thus, the C-terminal inhibitor CP, besides mimicking the biologic effects of the N-terminal inhibitor 17-AAG, may have another negative impact on Hsp90 function.

Given 17-AAG+CP was able to inhibit Hsp90 chaperone function, we assessed its inhibitory effect on client proteins in CML cells. Our result showed that 17-AAG+CP reduced Bcr-Abl and Raf protein levels corporately (Figure 2E and 2F).

Taken together, while both 17-AAG and CP could block Hsp90 chaperone function alone, the combination of 17-AAG and CP do not compete with each other in cells and have a synergistic effect on Hsp90 chaperone function inhibition.

### The combination of 17-AAG and CP suppresses leukemia cell proliferation synergistically

**Figure 3:**
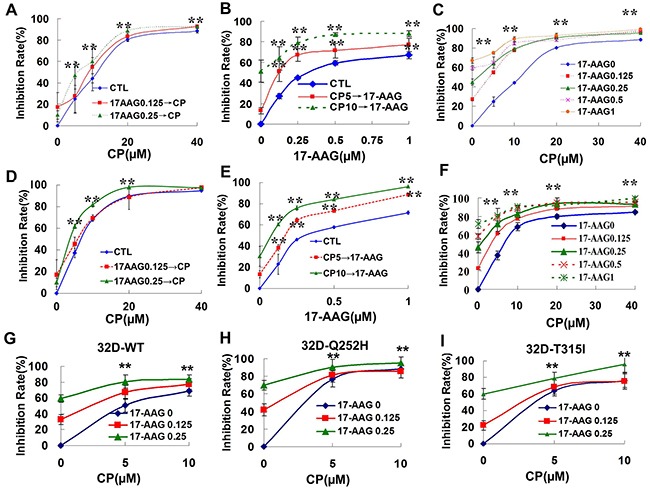
Synergistic cytotoxicity produced by 17-AAG and CP in Bcr-Abl-positive leukemia cells **A-C**. The survival of K562 cell was evaluated by MTT assay after the cells were treated with Hsp90 inhibitors. **A**. K562 cells were exposed to 0.125 or 0.25 μM 17-AAG for 3 h followed by exposure to CP at different concentration (5, 10, 20, 40 μM) for 48 h. ** p<0.01 vs control group. **B**. K562 cells were exposed to 5 or 10 μM CP for 3 h followed by exposure to 17-AAG at different concentrations (0.125, 0.25, 0.5, 1 μM) for 48 h. ** p<0.01 vs control group. **C**. K562 cells were exposed to 17-AAG+CP synchronously at different concentrations (17-AAG 0.125, 0.25, 0.5, 1 μM; CP 5, 10, 20, 40 μM) for 48 h. ** p<0.01 vs 17-AAG 0 group. **D-F**. The dose-response K562/G01 cell survival was evaluated by MTT assay after the cells were treated with Hsp90 inhibitors. **G-I**. The dose-response 32D-WT, 32D-Q252H, or 32D-T315I cell survival was determined after the cells were treated with 17-AAG+CP. ** p<0.01 vs 17-AAG 0 group.

Figure 3A, 3B, and 3C shows that the two drugs in combination produced a cooperative cytotoxic effect either via sequential or synchronous treatment. The equation for the conservative isobologram was used to calculate the combined index (CI) values shown in the fraction affected-CI plot constructed by median-effect method. Most of the CI values were <0.6 (Supplementary Figure 2B, 2D, 2F) after treatment with 17-AAG and CP, indicating that either combined 17-AAG and CP, synchronously or as a sequential treatment, produced synergistic cytotoxic effects in K562 cells. K562/G01 cells had a 15.2-fold resistance to imatinib compared with K562 cell line (Supplementary Figure 3A) [28]. Supplementary Figure 3B, 3C, 3D shows the combinational effects on imatinib-resistant cells K562/G01. The combinational therapy is also better than single treatment (Supplementary Figure 3E, 3F, 3G).

32D cells harboring the T315I or the Q252H site mutant of ABL kinase have some resistance to imatinib (Supplementary Figure 4). Figure 3G, 3H, and 3I shows that 17-AAG and CP were able to synergistically inhibit the proliferation of both the wild type and the site mutant of ABL kinase transferred 32D cells.

Therefore, the interaction between 17-AAG +CP and Hsp90 correlates with the cytotoxic activity.

### Combinational treatment with 17-AAG and CP induces apoptosis of imatinib-sensitive and imatinib-resistant CML cells

**Figure 4:**
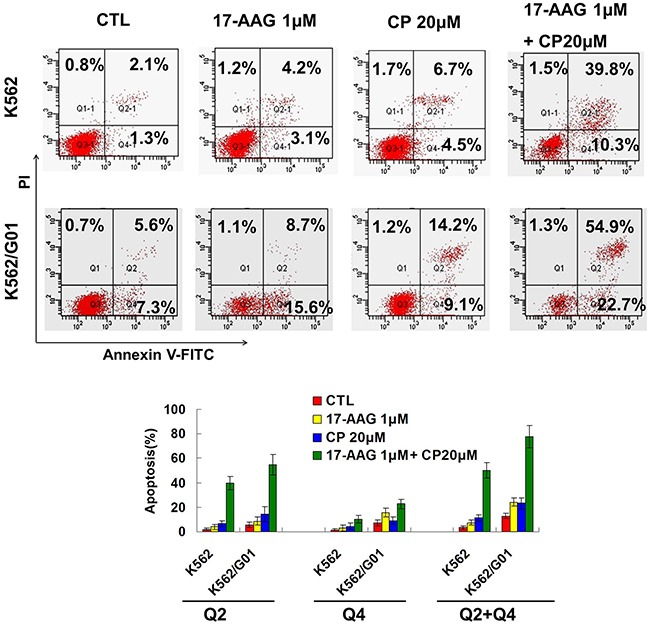
17-AAG and CP induce apoptosis synergistically in imatinib-sensitive and -resistant CML cells. After treating with 17-AAG 1μM and/or CP 20μM for 24 h, K562 and K562/G01 cells were incubated with Annexin V staining solution and then apoptosis were determined by flow cytometry.

We used annexin V-FITC/PI staining for the determination of apoptosis induced by Hsp90 inhibitors in CML cells. Consistent with the cellular proliferation assay results, apoptosis was induced synergistically in both the imatinib-sensitive and imatinib-resistant K562 cells (Figure 4).

### Combinational treatment with 17-AAG and CP blocks Bcr-Abl initiating signaling in leukemia cells

**Figure 5:**
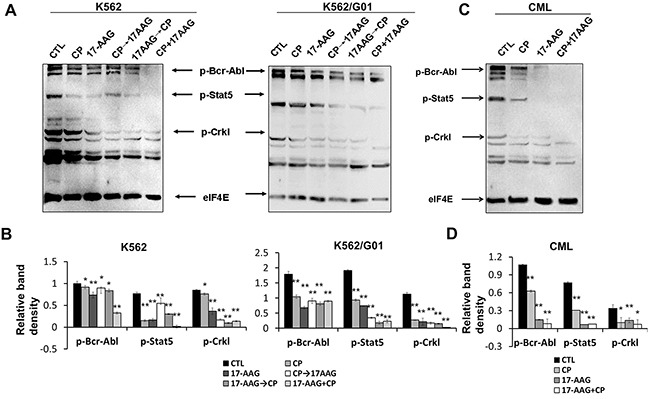
17-AAG and CP synergistically block the downstream pathway of Bcr-Abl in leukemia cells **A**. Treatment with 17-AAG, CP, 17-AAG+CP for 24 h, CP 3 h→17-AAG 24 h, 17-AAG 3 h→CP 24 h in cells retarded signal pathway initiated by Bcr-Abl. Thirty micrograms of cytosolic protein were resolved, transferred to a PVDF membrane, and then probed the level of p-c-Abl, p-Stat5, and p-CrkL. eIF4E served as input control. **B**. Band densities were measured and normalized to eIF4E. *p<0.05, ** p<0.01 vs control. **C**. Treatment with 17-AAG, CP, or 17-AAG+CP for 24 h has potent inhibition of Bcr-Abl initiating signaling in CD34+ cells from the bone marrow of CML patients. **D**. Band densities were normalized to eIF4E. *p<0.05, ** p<0.01 vs control. Note: 17-AAG: 1 μM 17-AAG; CP: 5 μM CP.

Because K562/G01 cells showed an increased level of Bcr-Abl proteins and an increased tyrosine kinase activity [28], we determined the effect of 17-AAG, CP, or 17-AAG+CP on the Bcr-Abl initiating signaling pathway. The results show that 17-AAG+CP showed synergistic inhibition of Bcr-Abl initiating signaling, including p-Bcr-Abl, p-Stat5, and p-Crkl in both imatinib-resistant K562/G01 cells and imatinib-sensitive K562 cells (Figure 5A, 5B). Moreover, 17-AAG+CP also caused potent inhibition of Bcr-Abl initiating signaling in CD34+ cells (Figure 5C, 5D).

### 17-AAG and CP suppress human leukemia progenitor/stem cells cooperatively

**Figure 6:**
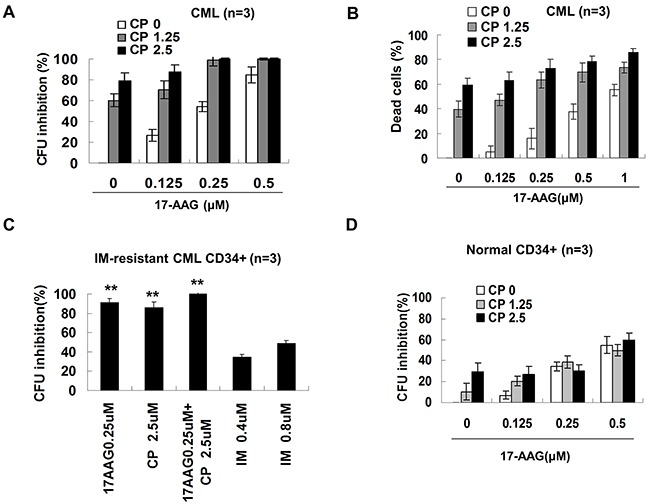
Effects of 17-AAG and CP on the survival of human progenitor cell **A**. CFU assay on progenitor cells from CML bone marrow. Enriched CML (n =3) CD34+ cells were treated with or without graded concentrations of 17-AAG and CP for 24 hours and further cultured for 14 days, then CFU cells were scored. **B**. Trypan blue exclusion measured the viable and dead CML CD34+ cells. After treating for 24 h, CD34+ cells were mixed with trypan blue and then the cell viability were determined. **C**. CFU assay ofimatinib-resistant CML CD34+ cells. ** p<0.01 vs IM 0.8 μM group. **D**. CFU assay on normal CD34+ cells. Values were expressed as the percentage compared to controls.

It is significant that treatment with 17-AAG 0.25 μM or 0.5 μM resulted in a marked suppression, by 54.3% or 84.8%, respectively, in colony formation of CD34+ CML cells (Figure 6A). CP at a 1.25 μM concentration inhibited colony-forming units (CFUs) to 60.35%. The combination of 17-AAG 0.25 μM and CP 1.25 μM resulted in a marked suppression up to 98.9%. The interaction between 17-AAG and CP was synergistic (CI < 1) according to the Chou–Talalay analysis (Supplementary Figure 5). The trypan blue exclusion has parallel results (Figure 6B). As expected, imatinib was not very sensitive to progenitor cells from this resistant patient. In contrast, Hsp90 inhibitors were able to suppress the growth of imatinib resistant progenitors efficiently (Figure 6C). After treatment with 0.25 μM 17-AAG or 1.25 μM CP, colony suppression was only 34.3% or 10.35%, respectively. Thus, CML progenitor cells were sensitive to Hsp90 inhibitors. Furthermore, Hsp90 inhibitors have less cytotoxic activity in normal a progenitor cells (Figure 6D).

Since LSCs are resistant to a variety of chemotherapeutic agents, including imatinib, we investigated the effects of the Hsp90 inhibitor co-treatment on LSCs by determining long term culture-initiating cells (LTC-IC), which represent primitive LSCs, by counting cobblestone area forming cells (CAFCs). CML MNCs (mononuclear cells) were treated with 17-AAG, CP or imatinib as single agents or in a combination treatment for 24 hours. The effects of Hsp90 inhibitors on LTC-IC are depicted in Figure 7A. The single agent 17-AAG has a minor inhibitory effect on LTC-IC. The high concentration of 17-AAG at 2 μM only moderately inhibits CAFC to 18.8%. In contrast, a CP 5 μM single treatment inhibited LTC-IC dramatically to 89.6%. Moreover, exposure to 17-AAG and CP together for 24 hours resulted in a slight increase in the suppression of CAFC compared with single treatment; however, this result was not statistically significant. To illustrate the effect of 17-AAG and CP on mature and primitive CML progenitor cells directly, apoptosis ratio of CD34+38+ and CD34+38- fractions was analyzed. 17-AAG and CP induced apoptosis in both t fractions (Figure 7B), demonstrating the activity of 17-AAG and CP in both quiescent and cycling cell populations.

## DISCUSSION

Hsp90 inhibitors are of interest because of their capacity of depleting several signaling molecules that are critical for the proliferation of many tumors. 17-AAG is currently in Phase II/III clinical evaluation for several cancers. However, although preclinical studies show promising antitumor activity *in vitro* and *in vivo*, 17-AAG has produced little clinical effect thus far as a single agent. This lack of activity has prompted studies related to combinational therapies.

Our present study evaluated the effects of the Hsp90 N-terminal inhibitor 17-AAG in combination with the Hsp90 C-terminal inhibitor CP in Bcr-Abl positive leukemia cells. Several studies have yielded somewhat conflicting reported results when 17-AAG and CP were combined in various tumor cell types. Vasilevskaya et al. initially reported additive effects when CP was combined with 17-AAG, although the combination was found to be antagonistic in some cell lines [29, 30]. In contrast, Bagatell et al. reported synergistic effects in neuroblastoma and osteosarcoma cell lines when GA was combined with CP via depletion of the Hsp90 client proteins AKT and the insulin-like growth factor 1 receptor (IGF1R) [31]. Likewise, our data show that the combination of 17-AAG and CP is synergistic *in vitro* using Bcr-Abl-positive leukemia cells, including imatinib sensitive K562 and resistant K562/G01 cells. Present studies show that co-treatment of the N-terminal inhibitor 17-AAG and the C-terminal inhibitor CP competed against the binding of Hsp90 to the N-terminal inhibitor GA more significantly than single agent treatment, using either a sequential or a simultaneous treatment. This result implied that 17-AAG and CP could bind to Hsp90 together but did not exhibit mutual antagonism. The Hsp90 protein, either as the purified protein without co-chaperones or the immunoprecipitated protein with co-chaperones from K562 cells or imatinib-resistant K562/G01 cells, can bind to 17-AAG and CP in a synchronized manner. Although the three domains of Hsp90 dynamically respond to client/co-chaperone protein binding and interact with each other, 17-AAG and CP would not antagonize each other on Hsp90 binding in the current model. In parallel to Hsp90 binding assay, two drugs in combination produced a cooperative cytotoxic effect (CI<1), either sequentially or as a synchronous treatment (Supplementary Figure 2 and 3).

Treatment with 17-AAG, CP, or 17-AAG+CP reduced the amounts of Hsp90 that were coprecipitated with Bcr-Abl. Compared with 17-AAG, CP did not induce the expression of Hsp70 in K562 and K562/G01 cells. Hsp70 contributes to Bcr-Abl–mediated resistance to apoptosis due to anti-leukemia agents [32]. Based on these findings, CP has an advantage over 17-AAG. Our results are consistent with Andrea K’s, who showed that a treatment with GA + CP revealed that CP suppresses up-regulation of HSF-1 transcription, causing decreased levels of stress-inducible proteins such as Hsp27 and Hsp70 [33].

Imatinib resistance includes *bcr-abl* gene amplification, and strategies for overcoming Bcr-Abl domain mutations will likely require inhibiting other molecular features of the Bcr-Abl protein. Hsp90 affects the maturation and stability of Bcr-Abl by chaperone function. Bcr-Abl point mutants isolated from patients with imatinib mesylate–resistant CML remain sensitive to Hsp90 inhibitors [34, 35]. Our results indicate 17-AAG+CP synergistically reduced the amounts of Hsp90 that were coprecipitated with Bcr-Abl, resulting in overcoming imatinib resistance.

CFU represents the growth of progenitors. CFU assays indicated 17-AAG+CP synergistically suppress the growth of leukemia progenitors.

LTC-IC is recognized as the most stringent assay to detect very primitive human hematopoietic stem cells *in vitro*. The addition of CP significantly reduced LTC-IC. In contrast, the single agent 17-AAG has moderate inhibitory effect on LTC-IC. Moreover, exposure to 17-AAG and CP together for 24 h inhibited CML LTC-IC significantly compared with a 17-AAG single treatment. These results indicated that the combination of 17-AAG and CP would eradicate CML leukemia stem cells efficiently.

In conclusion, our results indicate that cotreatment with N- and C-terminal Hsp90 inhibitors can destroy Hsp90 chaperone function synergistically in Bcr-Abl-positive human leukemia cells, containing imatinib-resistant CML cells,. Furthermore, either 17-AAG or CP has the capacity to suppress progenitors by CFU assay; however, only CP is able to inhibit leukemia stem cells significantly by LTC-IC assay, which implicates the combinational treatment is better than the single therapy and the former may suppress human leukemia cells in different differentiation states, including differentiated mature cells, progenitors, and LSCs (Figure 7D).

## MATERIALS AND METHODS

### Reagents

17-AAG and CP were bought from Sigma Chemical Co. (St. Louis, MO, USA). Biotin-Geldanamycin (Bio-GA) was purchased from Invitrogen (San Diego, CA, USA). 17-AAG or imatinib was dissolved in dimethylsulfoxide (DMSO) to obtain a stock solution with a concentration of 10 mM. In all the experiments, the control cells were incubated with DMSO alone. The final concentration of DMSO was maintained at 0.1% w/v. The antibodies used included anti-Hsp90 (ADI-SPA-845-D, Stressgen, PA, USA) and anti-p60^Hop^ (#4464), anti-p23 (#5128), anti-Hsp70 (#4873) and anti-Abl (#2862) antibodies, which were purchased from Cell Signaling Technology, Inc. (Danvers, MA, USA).

### Cell culture

K562 leukemia cell line and imatinib-resistant K562/G01 cell line were obtained and maintained as described previously [17]. The construction of 32D-WT, 32D-T315I and 32D-Q252H cell lines was described [36].

### Hsp90 binding assays [37]

Hsp90 protein (Stressgen, PA, USA) or cell lysates in lysis buffer (20 mM HEPES, pH7.3, 1 mM EDTA, 5 mM MgCl_2_, and 100 mM KCl) were treated with or without 17-AAG, CP, or 17-AAG+CP for 30 min at 4°C, and then the Hsp90 binding capacities were evaluated as described [37]. The obtained bands were analyzed using the Carestream Image Station.

### Sepharose 4B pulldown assay

Conjugated 17-AAG or/and CP-Sepharose 4B were prepared by incubating 17-AAG or/and CP with cyanogen bromide (CNBr)-activated Sepharose 4B (GE Healthcare, Sweden), and the pulldown assay was carried out as described [36].

### Cloning, expression, and purification of NHsp90

The recombinant vector NHsp90-pET28a was kindly provided by Professor Lianru Zhang, Xiamen University. Expression and purification of NHsp90 were described previously [36].

### Fluorescence quenching spectra of NHsp90

NHsp90 was prepared as described previously [36]. The fluorescence quenching spectra of NHsp90 was measured by maintaining NHsp90 at 5.0 M, and continuously increasing Hsp90 inhibitor solution from 5 μM to 50.0 μM. The measurement parameters were set as previously described [36].

### ATPase activity assay

Hsp90 ATPase activity was assayed by detecting the free inorganic phosphate (Pi) released from NHsp90. The measurements used a PiPer Phosphate Assay kit (P22061, Molecular Probes, Thermo Scientific, MA, USA) and performed according to manufacturer’s instruction. All the assays were performed in triplicate.

### Immunoprecipitation and western blotting analysis

The immunoprecipitation was performed by binding anti-Abl or anti-Hsp90 mAb to Dynabeads® Protein A/G beads and then incubating with cell lysates. The precipitation of proteins were eluted from the beads by boiling samples in SDS loading buffer, and the contents of Abl, Hsp90, Hsp70, p23, p60^Hop^ in precipitation were analyzed by western blotting as described [17]. The phosphorylated c-Abl, Stat5 and CrkL in cell lysates were detected by using PathScan^®^ Bcr/Abl Activity Assay cocktail (#5300, Cell Signaling Technology, Inc. MA, USA).

### MTT assays

The proliferation of cells was assessed by an MTT (3-(4,5-dimethylthiazol-2-yl)-2,5-diphenyltetrazolium bromide, Sigma Chemical Company, MO, USA) assay. Treated cells incubated with MTT solution and then the optical density was measured at 490nm.

### Annexin V detection for apoptosis

Collected cells were incubated with 100 μl Annexin-V staining solution (KeyGEN Biotech, Nanjing, China) for 15min at RT, and analyzed by flow cytometer (BD FACSCantoII, NJ, USA).

### CFU assays

Samples of human bone marrow were collected as previously described [17]. All human sample studies were approved by the Institutional Ethical Review Board of Fujian Medical University (Fuzhou, China). CD34+ cells were isolated by EasySep® Human Whole Blood CD34 Positive Selection Kit ((#18096, StemCell Technologies, Canada) and seeded in MethoCult® GF H4434 medium (StemCell Technologies, Canada) as previously described [38], the CFU cells were scored after 10-14 days.

### LTC-IC assay

MNC cells from CML bone marrow were seeded and maintained as previously described [38].5 weeks later *CAFCs* were counted.

### Statistical analyses

All statistical analyses were conducted with SPSS 19.0 Software. Data were examined using ANOVA with post hoc Student-Newman-Keuls test for multiple comparisons. An unpaired Student *t* test was used to determine the differences between single experimental and control groups. Differences with *P<0*.05 were considered significant.

## SUPPLEMENTARY MATERIALS



## References

[1] Neckers. L (2007). Heat shock protein 90: the cancer chaperone. J. Biosci.

[2] Travers J., Sharp S., Workman. P (2012). HSP90 inhibition: two-pronged exploitation of cancer dependencies. Drug Discov. Today.

[3] Neckers L., Workman. P (2012). Hsp90 molecular chaperone inhibitors: are we there yet?. Clin. Cancer Res.

[4] Workman P., Burrows F., Neckers L., Rosen. N (2007). Drugging the cancer chaperone HSP90: combinatorial therapeutic exploitation of oncogene addiction and tumor stress. Ann. N.Y. Acad. Sci.

[5] Neckers L., Ivy. S.P (2003). Heat shock protein 90. Curr. Opin. Oncol.

[6] Soti C., Racz A., Csermely. P (2002). A Nucleotide-dependent molecular switch controls ATP binding at the C-terminal domain of Hsp90. N-terminal nucleotide binding unmasks a C-terminal binding pocket. J. Biol. Chem.

[7] Soti C., Vermes A., Haystead T.A., Csermely. P (2003). Comparative analysis of the ATP-binding sites of Hsp90 by nucleotide affinity cleavage: a distinct nucleotide specificity of the C-terminal ATP-binding site. Eur. J. Biochem.

[8] Csermely P., Kajtar J., Hollosi M., Jalsovszky G., Holly S., Kahn C.R. (1993). ATP induces a conformational change of the 90-kDa heat shock protein (hsp90). J. Biol. Chem.

[9] Konopka JB, Watanabe SM, Witte ON (1984). An alteration of the human c-abl protein in K562 leukemia cells unmasks associated tyrosine kinase activity. Cell.

[10] Tauchi T., Broxmeyer. H.E (1995). BCR/ABL signal transduction. Int. J. Hematol.

[11] Daley GQ, Van Etten RA, Baltimore D (1990). Induction of chronic myelogenous leukemia in mice by the P210bcr/abl gene of the Philadelphia chromosome. Science.

[12] Deininger MW, Goldman JM, Melo JV (2000). The molecular biology of chronic myeloid leukemia. Blood.

[13] Li S, Ilaria RL, Million RP, Daley GQ, Van Etten RA (1999). The P190, P210, and P230 forms of the BCR/ABL oncogene induce a similar chronic myeloid leukemia-like syndrome in mice but have different lymphoid leukemogenic activity. J Exp Med.

[14] Soga S., Akinaga S., Shiotsu. Y (2013). Hsp90 inhibitors as anti-cancer agents, from basic discoveries to clinical development. Curr. Pharm. Des.

[15] Shiotsu Y., Soga S., Akinaga. S (2002). Heat shock protein 90-antagonist destabilizes Bcr-Abl/HSP90 chaperone complex. Leuk. Lymphoma.

[16] Peng C., Brain J., Hu Y., Goodrich A., Kong L., Grayzel D. (2007). Inhibition of heat shock protein 90 prolongs survival of mice with BCR-ABL-T315I-induced leukemia and suppresses leukemic stem cells. Blood.

[17] Wu L.X., Xu J.H., Zhang K.Z., Lin Q., Huang X.W., Wen C.X. (2008). Disruption of the Bcr-Abl/Hsp90 protein complex: a possible mechanism to inhibit Bcr-Abl-positive human leukemic blasts by novobiocin. Leukemia.

[18] Peng C., Li D., Li. S (2007). Heat shock protein 90: a potential therapeutic target in leukemic progenitor and stem cells harboring mutant BCR-ABL resistant to kinase inhibitors. Cell Cycle.

[19] Lu Z., Jin Y., Qiu L., Lai Y., Pan. J (2010). Celastrol, a novel HSP90 inhibitor, depletes Bcr-Abl and induces apoptosis in imatinib-resistant chronic myelogenous leukemia cells harboring T315I mutation. Cancer Lett.

[20] Gorre M.E., Mohammed M., Ellwood K., Hsu N., Paquette R., Rao P.N. (2001). Clinical resistance to STI-571 cancer therapy caused by BCR-ABL gene mutation or amplification. Science.

[21] von Bubnoff N., Schneller F., Peschel C., Duyster. J (2002). BCR-ABL gene mutations in relation to clinical resistance of Philadelphia-chromosome-positive leukaemia to STI571: a prospective study. Lancet.

[22] Carroll. Perl M (2011). BCR-ABL kinase is dead; long live the CML stem cell. J. Clin. Invest.

[23] Corbin A.S., Agarwal A., Loriaux M., Cortes J., Deininger M.W., Druker. B.J (2011). Human chronic myeloid leukemia stem cells are insensitive to imatinib despite inhibition of BCR-ABL activity. J. Clin. Invest.

[24] Passegue E., Wagner E.F., Weissman. I.L (2004). JunB deficiency leads to a myeloproliferative disorder arising from hematopoietic stem cells. Cell.

[25] Passegue E., Jamieson C.H., Ailles L.E., Weissman. I.L (2003). Normal and leukemic hematopoiesis: are leukemias a stem cell disorder or a reacquisition of stem cell characteristics?. Proc. Natl. Acad. Sci. U.S.A.

[26] Chen Y., Peng C., Sullivan C., Li D., Li. S (2010). Novel therapeutic agents against cancer stem cells of chronic myeloid leukemia. Anticancer Agents Med Chem.

[27] Chen Y., Hu Y., Zhang H., Peng C., Li. S (2009). Loss of the Alox5 gene impairs leukemia stem cells and prevents chronic myeloid leukemia. Nat Genet.

[28] Qi J., Peng H., Gu Z.L., Liang Z.Q., Yang. C.Z (2004). Establishment of an imatinib resistant cell line K562/G01 and its characterization. Zhonghua Xue Ye Xue Za Zhi.

[29] Vasilevskaya I.A., Rakitina T.V., O’Dwyer. P.J (2003). Geldanamycin and its 17-allylamino-17- demethoxy analogue antagonize the action of Cisplatin in human colon adenocarcinoma cells: differential caspase activation as a basis for interaction. Cancer Res.

[30] Vasilevskaya I.A., Rakitina T.V., O’Dwyer P.J. (2004). Quantitative effects on c-Jun N-terminal protein kinase signaling determine synergistic interaction of cisplatin and 17-allylamino-17-demethoxygeldanamycin in colon cancer cell lines. Mol. Pharmacol.

[31] Bagatell R., Paine-Murrieta G.D., Taylor C.W., Pulcini E.J., Akinaga S., Benjamin I.J. (2000). Induction of a heat shock factor 1-dependent stress response alters the cytotoxic activity of hsp90-binding agents. Clin. Cancer Res.

[32] Guo F., Rocha K., Bali P., Pranpat M., Fiskus W., Boyapalle S. (2005). Abrogation of heat shock protein 70 induction as a strategy to increase antileukemia activity of heat shock protein 90 inhibitor 17-allylamino-demethoxy geldanamycin. Cancer Res.

[33] McCollum Andrea K., Lukasiewicz Kara B., TenEyck Cynthia J., Lingle Wilma L., Toft David O (2008). Charles Erlichman. Cisplatin Abrogates the Geldanamycin-induced Heat Shock Response. Mol Cancer Ther.

[34] Gorre M.E., Ellwood Y. K., Chiosis G., Rosen N., Sawyers. C.L (2002). BCR-ABL point mutants isolated from patients with imatinib mesylate-resistant chronic myeloid leukemia remain sensitive to inhibitors of the BCR-ABL chaperone heat shock protein 90. Blood.

[35] Barnes D.J., De S., van Hensbergen P., Moravcsik E., Melo. J.V (2007). Different target range and cytotoxic specificity of adaphostin and 17-allylamino-17-demethoxygeldanamycin in imatinib-resistant and sensitive cell lines. Leukemia.

[36] Wu1 Lixian, Yu Jing, Chen Ruijia, Liu Yang, Lou Liguang, Wu Ying (2015). Dual inhibition of Bcr-Abl and Hsp90 by C086 potently inhibits the proliferation of imatinib-resistant CML cells. Clin Cancer Res.

[37] Thao Kamal L., Sensintaffar J., Zhang L., Boehm M.F., Fritz L.C., Burrows. F.J (2003). A high-affinity conformation of Hsp90 confers tumour selectivity on Hsp90 inhibitors. Nature.

[38] Li-xian Wu, Wu Ying, Chen Rui-jia, Liu Yang, Huang Li-sen, Lou Li-guang, Zheng Zhi-hong, Chen Yuan-zhong, Xu. Jian-hua (2014). Curcumin derivative C817 inhibits proliferation of imatinib-resistant chronic myeloid leukemia cells with wild-type or mutant Bcr-Abl in vitro. Acta Pharmacologica Sinica.

