# Supplementary material for: Hsp90 N- and C-terminal double inhibition synergistically suppresses Bcr-Abl-positive human leukemia cells
Source: Oncotarget. 2016 Dec 28;8(6):10025–36. doi: 10.18632/oncotarget.14324 (PMC5354638; doi:10.18632/oncotarget.14324)
Supplement: Supplementary file 1 [file oncotarget-08-10025-s001.pdf]

Hsp90 N- and C-terminal double inhibition synergistically suppresses Bcr-Abl-positive human leukemia cells

SUPPLEMENTARY MATERIAL

Chou–Talalay analysis

To determine the interactive effect between 17-AAG treatment and CP on CML CD34+ cells, Chou–Talalay analysis was performed as previously described [40]. The fixed ratios of 17-AAG and CP and mutually exclusive equations were used to determine the Combination Index (CI) according to the method of Chou–Talalay using Compusyn Software (Combosyn, Inc., NJ). Briefly, the combined dose–response curves were fitted to Chou–Talalay lines, which are derived from the law of mass action and described by the equation: $\log(f_a/f_u) = m \log(D) - m \log(Dm)$, in which f_a is the fraction affected, f_u is the fraction unaffected, D is the dose, D_m is the median-effect dose, and m is the coefficient signifying the shape of the dose–response curve. The CI values were calculated using the equation: CI = (D1/Dx1) + (D2/Dx2) + (D1) (D2)/[(Dx1) (Dx2)], where Dx1 and Dx2 are the 17-AAG and CP doses, respectively, which are required to achieve a particular f_a, and D1 and D2 are the doses of the 2 treatments (combined treatment) required for achieving the same f_a. CI < 1, CI = 1, and CI > 1 indicate synergistic, additive, and antagonistic interactions, respectively.

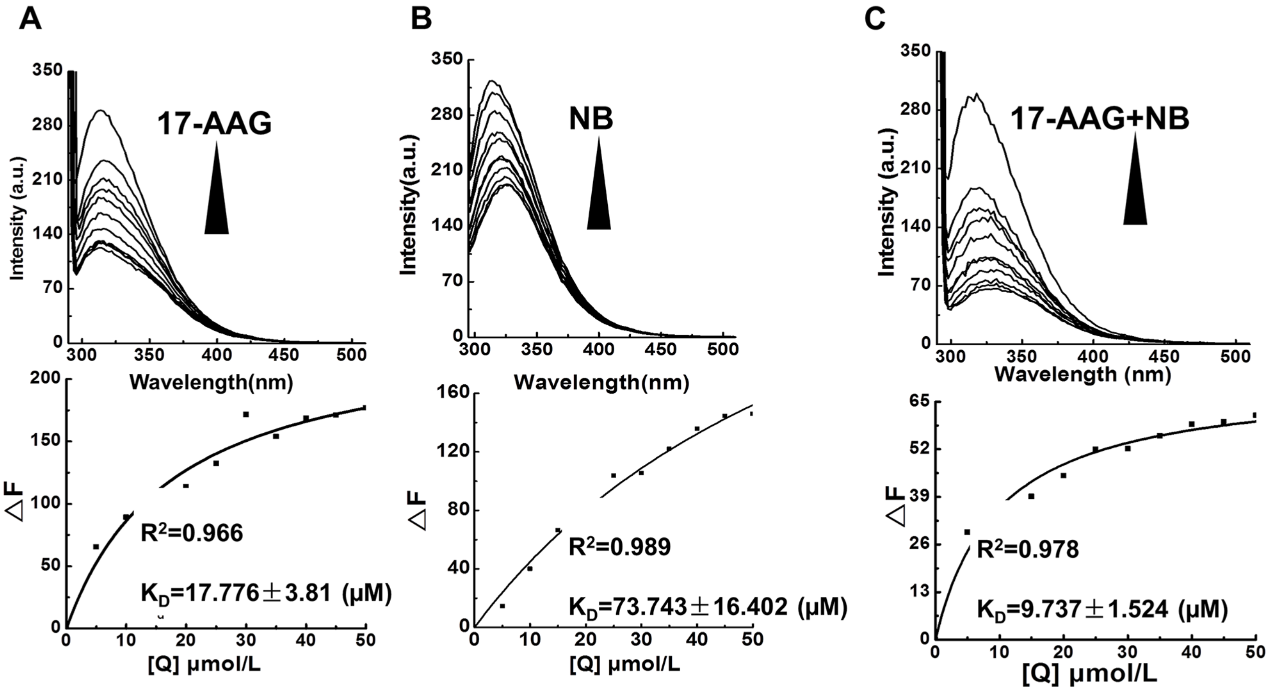

Supplementary Figure 1: The fluorescence quenching spectra of NHsp90 with 17-AAG, NOV, or 17-AAG+NOV. Upper panel: The concentration of NHsp90 was fixed at 5 μM, and the ratio of drugs vs NHsp90 was from 1:1 to 10:1. Lower panel: The variation tendency of ΔF.

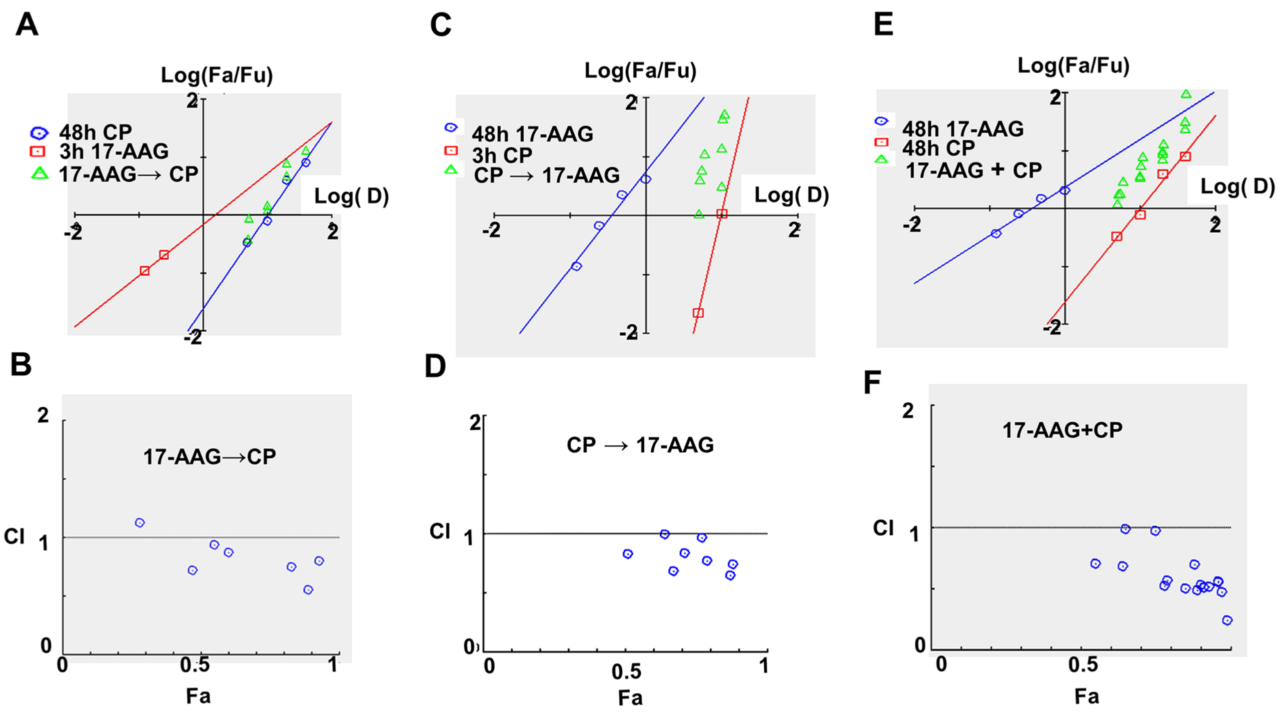

Supplementary Figure 2: A-B. Median-effect plot and fraction affected-CI plot of 17-AAG 3 h→CP 48 h were constructed by computer analysis of the data in Figure 3A using Compusyn software. CI values of <1 occurred at a wide range of inhibition levels, indicating synergy produced by the combination. **C-D.** Median-effect plot and fraction affected-CI plot of CP 3 h→17-AAG 48 h were constructed by computer analysis of the data in Figure 3B using Compusyn software. CI values of <1 occurred at a wide range of inhibition levels, indicating synergy produced by the combination. **E-F.** Median-effect plot and fraction affected-CI plot of 17-AAG+CP were constructed by computer analysis of the data in Figure 3C using Compusyn software. CI values of <1 occurred at a wide range of inhibition levels, indicating synergy produced by the combination.

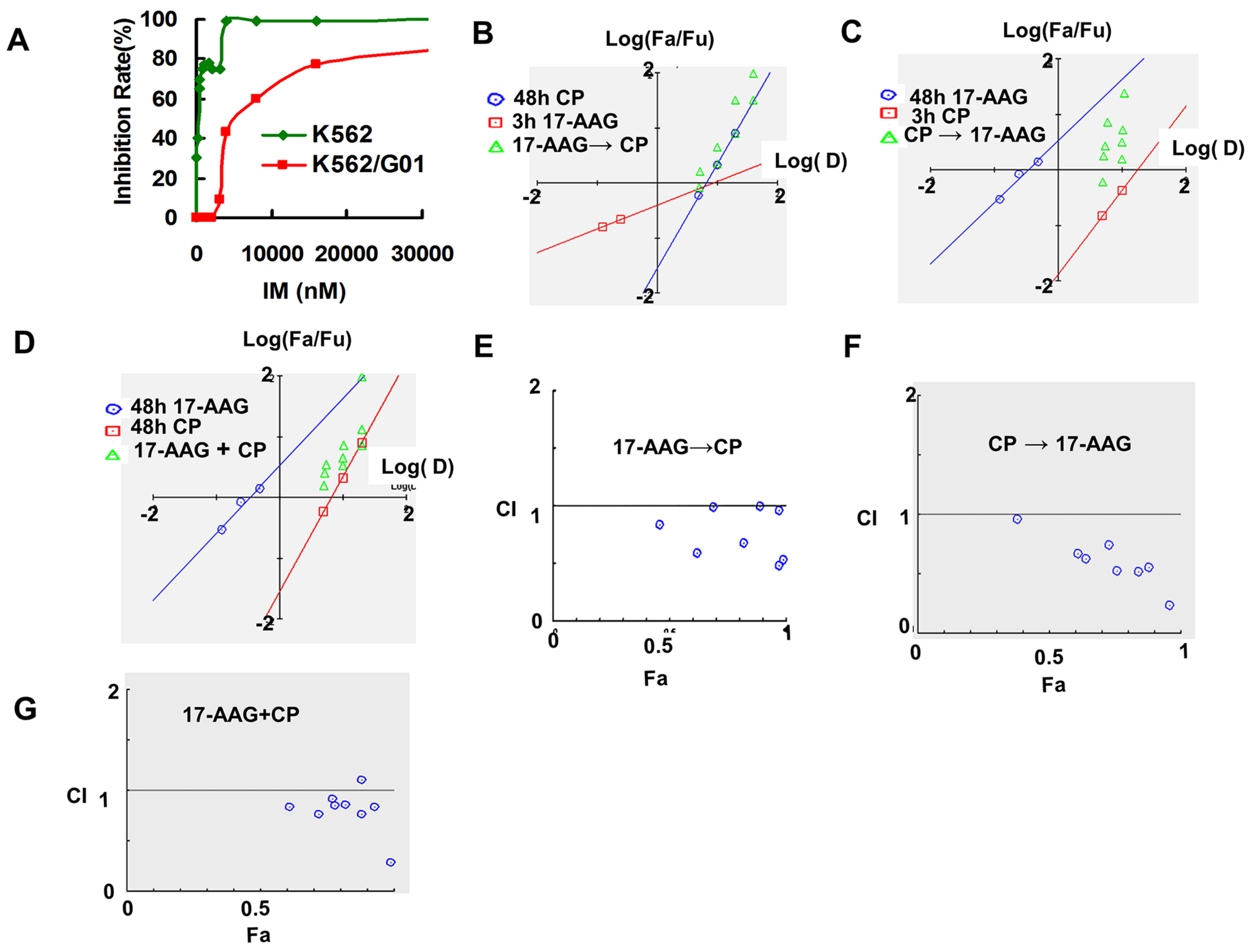

Supplementary Figure 3: A. The proliferation of K562 or K562/G01 cells in the presence of increasing concentrations of imatinib (0-32 000 nM). B. and **E.** Median-effect plot and fraction affected-CI plot of 17-AAG 3 h→CP 48 h were constructed by computer analysis of the data in Figure S3B using Compusyn software. CI values of <1 occurred at a wide range of inhibition levels, indicating synergy produced by the combination. **C.** and **F.** Median-effect plot and fraction affected-CI plot of CP 3 h→17-AAG 48 h were constructed by computer analysis of the data in Figure S3C using Compusyn software. CI values of <1 occurred at a wide range of inhibition levels, indicating synergy produced by the combination. **D.** and **G.** Median-effect plot and fraction affected-CI plot of 17-AAG+CP were constructed by computer analysis of the data in Figure S3D using Compusyn software. CI values of <1 occurred at a wide range of inhibition levels, indicating synergy produced by the combination.

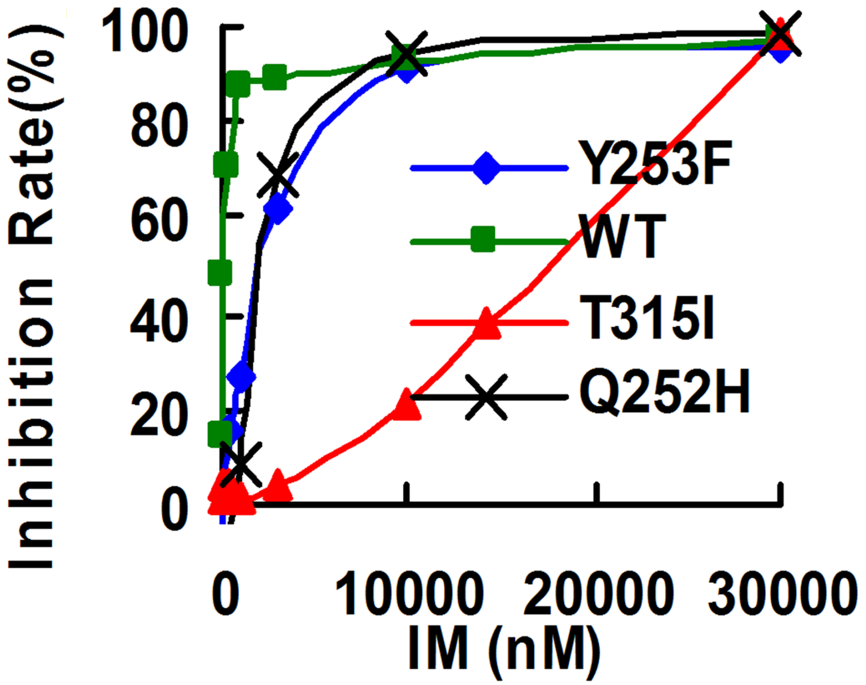

Supplementary Figure 4: The proliferation of 32D-WT, 32D-T315I, and 32D-Q252H cell lines in the presence of increasing concentrations of imatinib mesylate (0-30 000 nM).

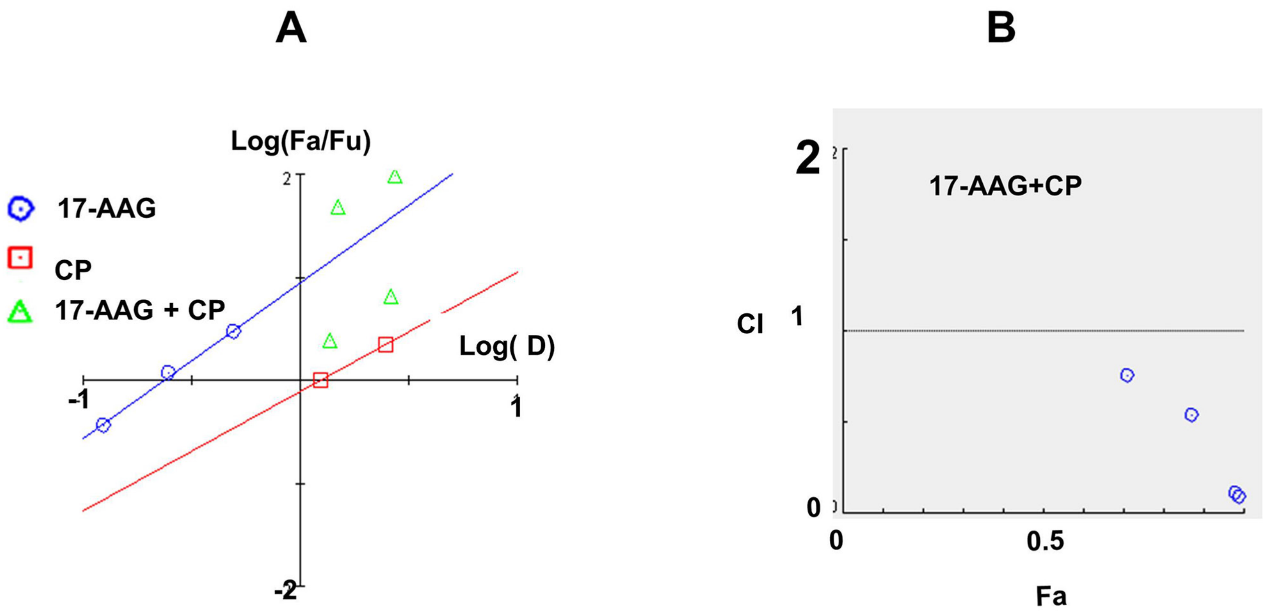

Supplementary Figure 5: Median-effect plot and fraction affected-CI plot of 17-AAG+CP were constructed by computer analysis of the data in Figure 6A using Compusyn software. CI values of <1 occurred at a wide range of inhibition levels, indicating synergy produced by the combination.
